# Diverse psychotropic substances detected in drug and drug administration equipment samples submitted to drug checking services in Toronto, Ontario, Canada, October 2019–April 2020

**DOI:** 10.1186/s12954-021-00585-2

**Published:** 2022-01-11

**Authors:** Kristy M. Scarfone, Nazlee Maghsoudi, Karen McDonald, Cristiana Stefan, Daniel R. Beriault, Ernest Wong, Mark Evert, Shaun Hopkins, Peter Leslie, Tara Marie Watson, Dan Werb, Toronto’s Drug Checking Service Working Group

**Affiliations:** 1 Centre On Drug Policy Evaluation, c/o Li Ka Shing Knowledge Institute of St. Michael’s Hospital, 30 Bond Street, Toronto, ON M5B1W8 Canada; 2 Leslie Dan Faculty of Pharmacy, University of Toronto, Toronto, ON Canada; 3 Institute of Health Policy, Management and Evaluation, University of Toronto, Toronto, ON Canada; 4 Clinical Laboratory and Diagnostic Services, Centre for Addiction and Mental Health, Toronto, ON Canada; 5 Department of Laboratory Medicine, St. Michael’s Hospital, Toronto, ON Canada; 6 Department of Laboratory Medicine and Pathobiology, University of Toronto, Toronto, ON Canada; 7 Toronto Public Health, Toronto, ON Canada; 8 Toronto Harm Reduction Alliance, Toronto, ON Canada; 9 Provincial System Support Program, Centre for Addiction and Mental Health, Toronto, ON Canada; 10 Division of Infectious Diseases and Global Public Health, University of California San Diego School of Medicine, La Jolla, CA USA

**Keywords:** Drug overdose, Fentanyl, Etizolam, Flualprazolam, Synthetic cannabinoid

## Abstract

**Background:**

The overdose crisis has generated innovative harm reduction and drug market monitoring strategies. In Toronto, Ontario, Canada, a multi-site drug checking service (DCS) pilot project was launched in October 2019. The project provides people who use drugs with information on the chemical composition of their substances, thereby increasing their capacity to make more informed decisions about their drug use and avoid overdose. DCS also provides real-time market monitoring to identify trends in the unregulated drug supply.

**Methods:**

Sample data were obtained through analyses of drug and used drug administration equipment samples submitted anonymously and free of charge to DCS in downtown Toronto from October 10, 2019, to April 9, 2020, representing the first six months of DCS implementation. Analyses were conducted in clinical laboratories using liquid chromatography- and/or gas chromatography-mass spectrometry (LC–MS, GC–MS) techniques.

**Results:**

Overall, 555 samples were submitted, with 49% (271) of samples that were found to contain high-potency opioids, of which 87% (235) also contained stimulants. Benzodiazepine-type drugs were found in 21% (116) of all samples, and synthetic cannabinoids in 1% (7) of all samples. Negative effects (including overdose, adverse health events, and extreme sedation) were reported for 11% (59) of samples submitted for analysis.

**Conclusions:**

Toronto’s DCS identified a range of high-potency opioids with stimulants, benzodiazepine-type drugs, and a synthetic cannabinoid, AMB-FUBINACA. This information can inform a range of evidence-informed overdose prevention efforts.

## Introduction

The opioid overdose crisis is worsening throughout much of North America, particularly in Canada and the United States [1, 2]. Between January 2016 and March 2020, more than 16,364 Canadians died from apparent opioid-related overdoses [3]. Recent estimates suggest that 1 in 6 deaths of Ontarians aged 25–34 years is related to opioid overdose [4]. Moreover, the incidence of fatal overdose has increased in Ontario since the imposition of COVID-19 restrictions in March 2020 [5].

Public health alerts from Toronto Public Health exemplify patterns in adulteration of the unregulated street supply of substances in the downtown core over the course of the last decade. Between 2004 and 2013, Toronto Public Health reported a 41% increase in the reported number of fatal overdoses in Toronto, increasing from 146 to 206 [6]. From this time and until 2016, the unregulated drug supply was generally known to contain heroin by majority, and fentanyl as an emerging adulterant. Since 2016, the number of lives lost to fatal overdoses, and/or serious complications related to non-fatal overdoses, has only risen. Incidence of serious complications associated with opioid overdose has also increased by 67% from 2010 to 2019, 10% of whom died in the year following discharge [7]. It is now understood by service users and front-line support services that fentanyl and related high potency opioids are no longer a mere minor adulterant in the unregulated street supply of opioids but have now replaced what was largely a heroin market in 2016 as the current opioid majority and leads as the most commonly present opioid in accidental overdose deaths in Ontario [8, 9].

The dynamic uncertainty in the unregulated drug market has generated innovative harm reduction approaches in Canada, including the implementation of drug checking services (DCS) tailored for structurally vulnerable people who use drugs [10]. Originating in California amid the counterculture movement in the 1960s and early 1970s [11], and having later gained popularity in the 1990s in European nightlife and dance settings [12], DCS provide chemical analysis of substances to service users, helping them to make informed drug use decisions, while contributing data to drug market monitoring [13].

A multi-site DCS pilot was launched in Toronto, Ontario, Canada in October 2019 [14]. Intake sites are co-located with supervised consumption services at multiple harm reduction agencies. The project prioritizes providing structurally vulnerable (i.e., socially and economically marginalized) people who use drugs with information about the composition of their substances, thereby increasing their capacity to avoid overdose.

This report presents early trends of samples analyzed within the first six months of DCS implementation in Toronto (October 10, 2019–April 9, 2020). We sought to identify the prevalence of high-potency opioids in the unregulated drug supply and to identify noteworthy combinations thereof with stimulants, benzodiazepine-type drugs, and synthetic cannabinoids. We also present data on reported negative effects of samples (e.g., overdose).

## Methods

In October 2019, Toronto’s Drug Checking Service launched DCS in downtown Toronto in collaboration with toxicology laboratories at the Centre on Addiction and Mental Health and St. Michael’s Hospital, Toronto Public Health, and community partners. The protocol and rationale for the evaluation of Toronto’s DCS has previously been described [10]. In brief, we are evaluating the impact of Toronto’s DCS on overdose and related risk behaviors among service users and its capacity to identify trends in the chemical composition of Toronto’s unregulated drug supply. The results presented herein were obtained through chemical analyses of samples submitted anonymously and free of charge to DCS by a service user (i.e., the person who used or plans to use the substance, someone on their behalf, or other interested parties such as drug sellers), or a staff member at the harm reduction site on behalf of a service user. The submitted sample is assigned an anonymous and unique identifier code. Samples are analyzed at the Centre for Addiction and Mental Health or at St. Michael’s Hospital using liquid chromatography- and/or gas chromatography-mass spectrometry (LC–MS, GC–MS) techniques, which are the gold standards in forensic drug analysis [15].

The analytic results are supplemented by data from surveys administered during sample collection, which solicit information on the expected drug, whether drug administration equipment submitted for analysis was reused, and negative effects post-use. Upon submission, the individual is asked “What was this sample purchased as (?),” which is reported as the drug that the individual expected to be present in the sample based on information provided at the time of purchase. The service user is also asked “Are you aware of this sample being associated with an overdose or adverse effect (?)”. An overdose is self-reported by the service user or by a staff member at the harm reduction site on behalf of the service user.

Results are provided within one to two business days. The service user may retrieve the analysis results through the unique identifier code assigned upon submission. Analysis results are shared with the individual alongside tailored harm reduction strategies. Aggregated results are publicly shared on a dedicated website bi-weekly (drugchecking.cdpe.org) [9], on bulletin boards at harm reduction agencies, on social media, through clinical and harm reduction listservs, and alerts are distributed by Toronto Public Health.

## Results

**Fig. 1:**
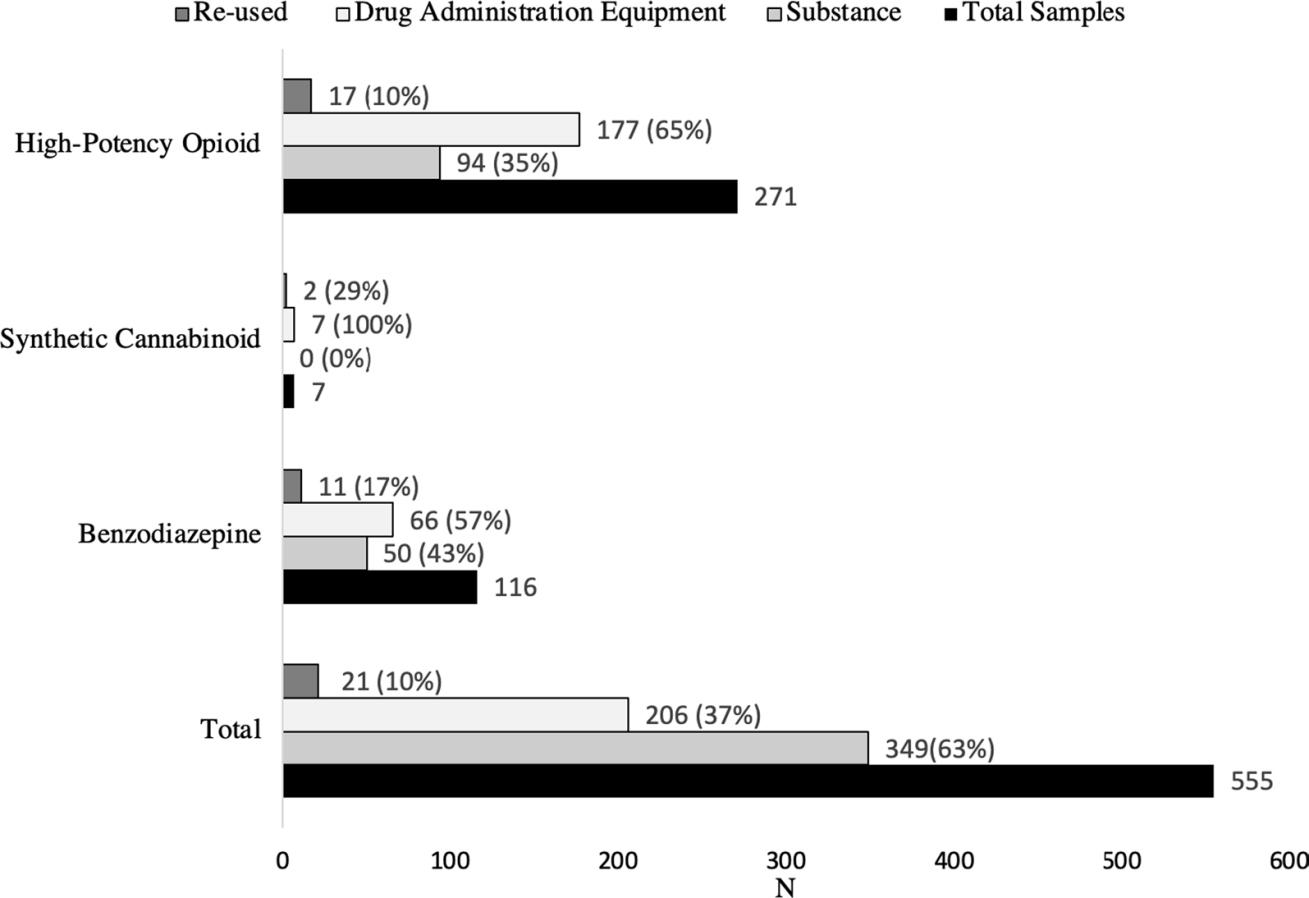
Graphical depiction of sample types stratified by category of drug detected (i.e., benzodiazepine, synthetic cannabinoid, or high-potency opioid) compared to total number of samples in that category (N = 555)

Overall, 555 samples were submitted for analysis by DCS, among which 62% (n = 344) were substances (i.e., liquid, pill, powder) and 38% (n = 211) drug administration equipment (i.e., cooker, filter, or leftover liquid from a used syringe), 10% (n = 21) of which were reportedly reused. The number of samples stratified by type and category of drug detected are presented in Fig. 1. Herein, data is presented by sample type, as drug administration equipment may contain remnants of chemicals from multiple uses and therefore may be less reliable than submitted substance samples with respect to adulteration. Nevertheless, both are important identifiers of what is currently circulating in the unregulated drug supply in downtown Toronto during the defined study period.

### Expected opioids and detected contents

**Fig. 2:**
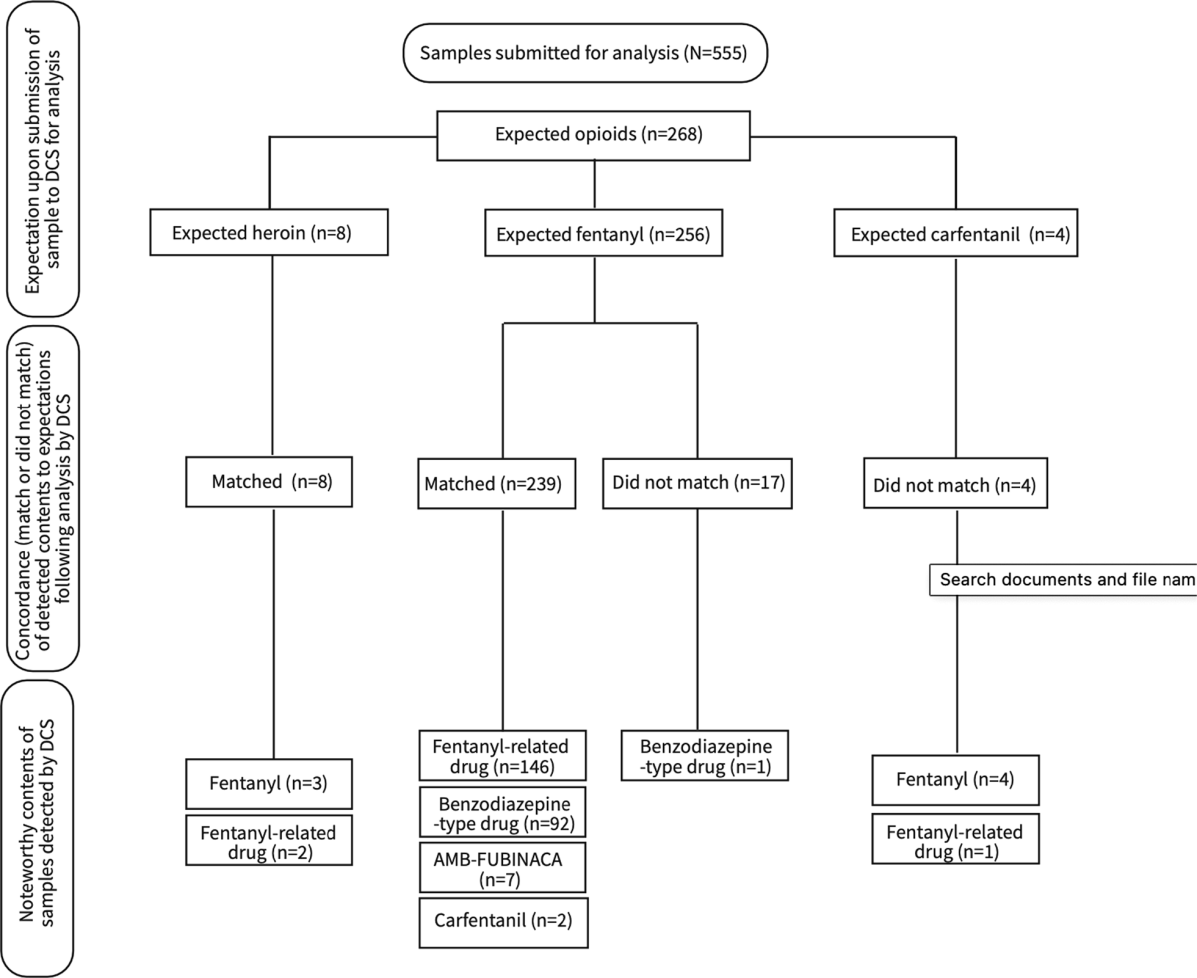
A breakdown of high-potency opioids, benzodiazepine-type drugs, and synthetic cannabinoids found in samples expected to be heroin, fentanyl, and carfentanil (n = 268)

Of all samples submitted for analysis, 48% (n = 268) were expected to be the following opioids: carfentanil, fentanyl, or heroin. Fentanyl was expected most often, in 96% (n = 256) of the expected opioid samples. Figure 2 depicts the detected drugs in these expected opioid samples, including unexpected high-potency opioids, unexpected benzodiazepine-type drugs, and unexpected synthetic cannabinoids. Each sub-category of drugs detected are presented below in the context of being detected with high-potency opioids.

Furthermore, a sensitivity and specificity analysis with regards to concordance between expected and detected samples for all opioids, fentanyl, and heroin respectively are presented below in Table 1. Of note is the discordance evident for samples where heroin was detected by laboratory analysis but was not expected upon submission. Of these samples, 95% (n = 106) were expected to be fentanyl.

**Table 1:** Positive predictive values (PPV), negative predictive values (NPV), sensitivity and specificity are reported for concordance of expected and detected opioids, fentanyl, and heroin in samples submitted to DCS in Toronto, ON October 2019 to April 2020 (N = 555)

Opioid	Detected	Not detected	Total	PPV	94.08% (90.92, 96.18)
Expected	270	17	287	NPV	91.79% (88.2, 94.36)
Not expected or unknown	22	246	268	Sensitivity	92.47% (88.82, 95.22)
Total	292	263	N = 555	Specificity	93.54% (89.85, 96.19)

Fentanyl	Detected	Not detected	Total	PPV	93.31% (89.91, 95/62)
Expected	251	18	256	NPV	93.01% (89.71, 95.31)
Not expected or unknown	20	266	299	Sensitivity	92.62% (88.83, 95.43)
Total	271	284	N = 555	Specificity	93.66% (90.17, 96.20)

Heroin	Detected	Not detected	Total	PPV	100%
Expected	8	0	8	NPV	79.52% (78.74, 80.29)
Not expected	112	435	547	Sensitivity	6.67% (2.92, 12.71)
Total	120	435	N = 555	Specificity	100% (99.16, 100)

### Expected stimulants and detected contents

**Fig. 3:**
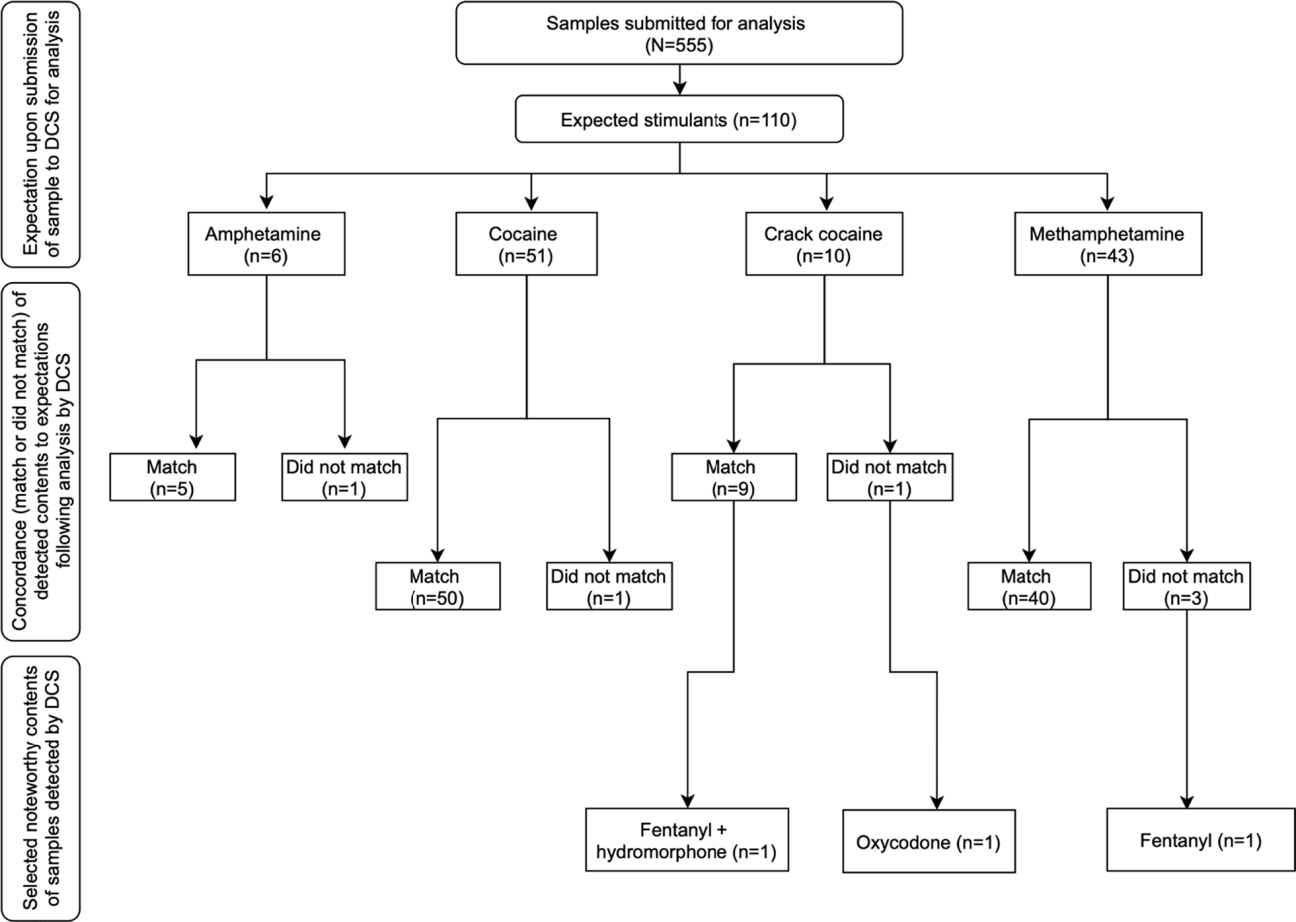
A breakdown of unexpected fentanyl and related opioid drugs detected among the associated expected stimulant samples submitted to DCS (n = 110). *MDA: 3,4-Methylenedioxyamphetamine. **MDMA: 3,4-Methylenedioxymethamphetamine

Of all samples submitted for analysis, 20% (n = 110) were expected to be the following stimulants: amphetamine, cocaine, crack cocaine, or methamphetamine. Most samples were submitted as substances (n = 98). Noteworthy findings post-analysis include detection of fentanyl, hydromorphone, and oxycodone unexpectedly in 4% (n = 4) of expected stimulant samples. Fentanyl and hydromorphone were both detected unexpectedly in one expected crack cocaine sample submitted as drug administration equipment. Oxycodone was detected unexpectedly in a second expected crack cocaine sample submitted as a substance (note this sample did not contain cocaine—oxycodone was the sole compound found). Fentanyl was also detected unexpectedly in one expected methamphetamine sample submitted as a substance, which was reported as being associated with an overdose (note this sample did not contain methamphetamine—fentanyl was the sole compound found). These findings are summarized in Fig. 3.

### Presence of stimulants with high-potency opioids

Of all samples submitted for analysis, 49% (n = 271) contained a high-potency opioid (i.e., fentanyl, fentanyl-related drugs, carfentanil). In 87% (n = 235) of these samples where a high-potency opioid was detected, one or more stimulants were found in combination, including caffeine, cocaine, or methamphetamine. Of this fraction of samples, a high-potency opioid (i.e., fentanyl, carfentanil) was expected in 210 samples, or 89%. The most frequent combinations detected were fentanyl with caffeine (n = 215), with cocaine (n = 57), and with methamphetamine (n = 43). Caffeine was the most commonly co-detected stimulant in 79% (n = 215). A stimulant was not expected in 97% of samples where a high potency opioid was detected with at least one stimulant. Drug administration equipment represents 67% (n = 181) of these samples.

### Presence of benzodiazepine-type drugs

Of all samples submitted for analysis, 21% (n = 116) contained at least one benzodiazepine-type drug. Of these samples, 67% (n = 78) contained only one benzodiazepine and 33% (n = 38) contained two or more. Samples were submitted as a substance in 43% (n = 50), 4 of which were reported to be in pill form (e.g., described as “light green pill”, “white pill”, or “pill”). Other substance sample characteristics as described by the service user were “purple/blue” substances (n = 17), “clear” substance (n = 1), a “green powder” (n = 1), “pink to blue when cooked” (n = 1), and “yellow” (n = 1). The remaining samples were submitted as drug administration equipment (n = 66) whose visual characteristics were not described.

Majority of the detected benzodiazepines were found unexpectedly in submitted samples; only 13% (n = 15) were expected benzodiazepines. Fentanyl was expected in 80% (n = 93), and various other drugs in the remaining 7% (n = 8). Of the samples where fentanyl was expected and benzodiazepines were detected, 65% (n = 60) were submitted as drug administration equipment and 35% (n = 33) as substances.

The primary benzodiazepine-type drugs detected were etizolam (n = 95), flualprazolam (n = 46), alprazolam (Xanax) (n = 9), flubromazolam (n = 4), and meclonazepam (n = 1). Samples containing benzodiazepine-type drugs account for 41% (n = 24) of all samples associated with a negative effect during the study period, 67% (n = 16) submitted as drug administration equipment and 33% (n = 8) as substance-type samples (i.e., an overdose was implicated in 67% (n = 16) of the samples where a benzodiazepine-type drug was detected 31% (n = 5) as substance-type sample and 69% (n = 11) as drug administration equipment sample).

### Presence of synthetic cannabinoids

Of all samples submitted for analysis, 1% (n = 7) contained a synthetic cannabinoid, all of which were expected to be fentanyl. AMB-FUBINACA was the only synthetic cannabinoid detected.

### Negative effects

Survey responses indicate that 11% (n = 59) of all samples submitted for analysis were associated with negative effect. Of these samples, survey responses report that: 63% (n = 37) were implicated in an overdose, 29% (n = 17) were associated with adverse health events, and 8% (n = 5) were associated with extreme sedation.

## Discussion

Across a six-month period in Toronto, a large proportion of samples submitted to DCS contained high-potency opioids presenting with unexpected stimulants. We also detected various benzodiazepine-type drugs and a synthetic cannabinoid, AMB-FUBINACA.

In the results presented herein, DCS provide further evidence that the current opioid market in downtown Toronto, Ontario, is saturated with fentanyl both appearing as an adulterant but also as a predominant expected substance upon sample submission. This prevalence of fentanyl and related high-potency opioids is noteworthy given that they carry increased risk for overdose. In particular, when stimulants are adulterated with high-potency opioids such as fentanyl and related drugs, the risk for harms, including overdose, increases. This pattern of adulteration has contributed to increasing polydrug overdose mortality [16]. Caffeine was the most common stimulant co-presenting with high-potency opioids, which is consistent with other settings [17].

The detection of various benzodiazepine-type drugs is significant given the risk of overdose that they present, especially when unexpected and combined with opioids. Benzodiazepines elicit sedative and anxiolytic effects that, when combined with other central nervous system depressants, may amplify cardiovascular and respiratory depression and contribute to overdose severity [18]. Compounding this issue is the lack of reliable and effective antagonist therapies to reverse benzodiazepine overdose outside of clinical settings [19]. Additionally, increased use of benzodiazepines may increase tolerance and the potential for withdrawal, especially if individuals are not aware that they are using these substances. This issue therefore demands greater urgency, as it appears to be contributing to overdose mortality in Toronto and elsewhere [20]. For instance, on February 26, 2020, a notable spike in overdoses (16 in seven hours) in Toronto was associated with drug samples containing large proportions of flualprazolam along with fentanyl and caffeine [21].

We also detected AMB-FUBINACA, an ultra-potent synthetic cannabinoid that can be up to hundreds of times more potent than D9-tetrahydrocannabinol [22]. Synthetic cannabinoid exposure may cause adverse effects including neuropsychiatric, cardiovascular, and renal impairment [23], but the effects of co-administration of AMB-FUBINACA and other central nervous system depressants (e.g., benzodiazepines, opioids) remain largely unknown. Antagonist therapies for synthetic cannabinoid intoxication have yet to be elucidated, and supportive measures relevant to symptom management are recommended (e.g., intravenous hydration, antipsychotic in the cases of acute neuropsychiatric impairment, etc.) [24]. It is therefore critical to share information on their presentation in unregulated drug markets with people who use drugs, harm reduction workers, and clinicians.

DCS serve as a tool to monitor and alert the public on current drug supply trends and potential health harms. In aggregate forms, this information can help front line support (i.e., paramedics, service providers) to appropriately respond to impending cases of overdose. For example, if benzodiazepines or synthetic cannabinoids are recognized as a common adulterant to opioid samples (and thereby taken unknowingly), providing robust doses of naloxone may not be sufficient in rousing the individual, and supportive measures related to benzodiazepine or synthetic cannabinoid overdose may be applied [18, 24]. Beyond providing information to the public, when results of the chemical analysis of submitted samples are disseminated to service users themselves, they are bolstered with tailored harm reduction strategies relevant to their personal substance use (e.g., protective behaviours such as not using alone, carrying naloxone, using safe consumption sites, using a smaller “test” dose, or discarding toxic substances). These personalized strategies can reduce harms associated with use of substances in discordance with what they were sold as or anticipated to be.

There are limitations to the capacity of Toronto’s DCS to provide a comprehensive analytical assessment of the unregulated drug supply. Inherent limitations of mass spectrometry analysis include accessibility, cost, destruction of the sample during analysis, contaminants obscuring data outputs, the speed at which results can be provided to service users, and the inability to detect certain non-drug compounds (e.g., metals, pesticides, inorganic salts, sugars) [15]. With regards to sample composition, the results may not represent the entire supply that samples are taken from, and reused drug administration equipment may contain substances from multiple uses and considered in interpretation of DCS data on co-presentation of samples of this type.

## Conclusions

This report briefly highlights the presence of highly-potent substances and combinations thereof in Toronto’s unregulated drug market. Timely data and public health alerts are critical to inform people who may experience health harms from drug use and demonstrate the value of DCS for drug market monitoring. Examples of such alerts were issued by Toronto Public Health regarding noteworthy trends in Toronto’s unregulated drug supply [20].

However, DCS alone are insufficient to prevent overdose and mortality arising from an unregulated drug supply, especially among persons without access to medication-assisted treatment for drug dependence or other standard-dose regimens as a result of drug policies (e.g., drug criminalization) that amplify social marginalization [25]. Quantifying the persisting contaminated drug supply and addressing it with policy solutions such as safer supply or other standard-dose modalities is therefore urgently needed [26].

As the global COVID-19 pandemic continues, drug trafficking patterns and supply are likely to remain dynamic [22]. This increases the need to sustain and expand DCS to prevent overdose and to monitor drug market fluctuations.

## Data Availability

A selection of the data presented herein is available online at: https://drugchecking.cdpe.org/.
